# CSGL: chemical synthesis graph learning for molecule representation

**DOI:** 10.1093/bioinformatics/btaf355

**Published:** 2025-06-19

**Authors:** Anchen Li, Elena Casiraghi, Juho Rousu

**Affiliations:** Department of Computer Science, Aalto University, Espoo 02150, Finland; Department of Computer Science, Aalto University, Espoo 02150, Finland; AnacletoLab, Dipartimento di Informatica “Giovanni degli Antoni”, University of Milan, Milan 20133, Italy; Environmental Genomics and Systems Biology Division, Lawrence Berkeley National Laboratory, Berkeley, CA 94720, United States; ELLIS, European Laboratory for Learning and Intelligent Systems, Milan Unit (University of Milan), Milan 20133, Italy; Department of Computer Science, Aalto University, Espoo 02150, Finland

## Abstract

**Motivation:**

Molecule representation learning (MRL) translates molecules into a real vector space, serving as input to downstream tasks in biology, chemistry, and computer science. This article introduces a chemical synthesis graph learning (CSGL) framework, which enhances MRL by considering both the atomic structures of molecules and their roles in chemical reactions through a hierarchical graph representation. Specifically, molecules are first modeled based on their molecular graphs, which capture atomic-level structural information. They are then further refined using a chemical synthesis graph, where nodes represent reactant and product molecule sets, and edges encode chemical transformations between reactants and products (e.g. changes in molecular structures). CSGL optimizes molecular embeddings of reactant and product nodes in a fashion that ensures the embeddings conform to a chemical balance constraint.

**Results:**

Experimental results show that our method CSGL achieves strong performance on a variety of tasks, including product prediction, reaction classification, and molecular property prediction.

**Availability and implementation:**

https://github.com/li-2023/CSGL.

## 1 Introduction

The intersection of molecular science and machine learning has attracted considerable interest from researchers in both fields. Molecular representation learning (MRL) is key in linking these fields (Guo *et al.* 2022, Su *et al.* 2022, Wang *et al.* 2022b). MRL transforms molecules into the low-dimensional embeddings that preserve crucial molecular information. These embeddings can serve as input features for various biological and chemical applications, including reactant prediction (Wang *et al.* 2022a), molecule generation (Mahmood *et al.* 2021), retrosynthesis planning (Segler *et al.* 2018), and molecule property prediction (Nguyen *et al.* 2024, Ding *et al.* 2025).

MRL approaches can be broadly divided into two types (Wang *et al.* 2022a, Li *et al.* 2024). The first category (Chithrananda *et al.* 2020, Fabian *et al.* 2020) uses SMILES strings as input and apply natural language processing (NLP) models [e.g. Transformers (Vaswani *et al.* 2017) and BERT (Devlin *et al.* 2018)] to learn molecular representations. While effective, these methods often struggle to consider the molecular structure. This is because the SMILES represents molecules as 1D linear strings based on the traversal order of the molecular graph. As a result, atoms close together in the SMILES string might be far apart in the actual molecule, which may mislead NLP methods that depend on the token position. The second category, structure-based methods, subdivides into fingerprint-based and graph neural network (GNN)-based. The former considers molecular substructures as words and molecules as sentences, and uses NLP models to learn molecule embeddings (Rogers and Hahn 2010, Kearnes *et al.* 2016, Jaeger *et al.* 2018). Nevertheless, they often struggle to capture the substructure importance and are difficult to train end-to-end (Wang *et al.* 2022a). In contrast, GNN-based methods (You *et al.* 2020, Xu *et al.* 2021) address these limitations and are further advanced by utilizing contrastive learning, which extracts the self-supervision signals from molecular structures. Despite these advances, they mainly focus on developing GNN architectures for modeling molecules and may not effectively integrate domain-specific knowledge.

To enhance the MRL task, we leverage the domain-specific knowledge of chemical reactions, which are represented by equations showing reactant molecules on the left side and product molecules on the right side. In recent years, related works, such as MolR (Wang *et al.* 2022a) and our previously proposed RXGL (Li *et al.* 2024) approaches, also leverage reactions for MRL. Specifically, MolR optimizes the molecular representation by imposing that, for each reaction, the sum of the reactant molecule representations equals the sum of the product molecule representations. However, this approach oversimplifies the inherent nature of chemical reactions and neglects the transformations that occur between reactants and products. RXGL method first establishes the relation between individual reactant molecules and individual product molecules in reactions, and then learns molecular representations based on this relation. In addition, RXGL introduces a memory network to learn the underlying implicit transformations occurring in reactions. However, this molecule-level relationship might introduce noise, potentially reducing the accuracy of molecular modeling. Moreover, RXGL fails to account for the real features in reaction transformations, such as bond number alterations (bond breaking and forming), which lacks effective guidance in modeling and optimizing molecular representations.

**Figure 1.**
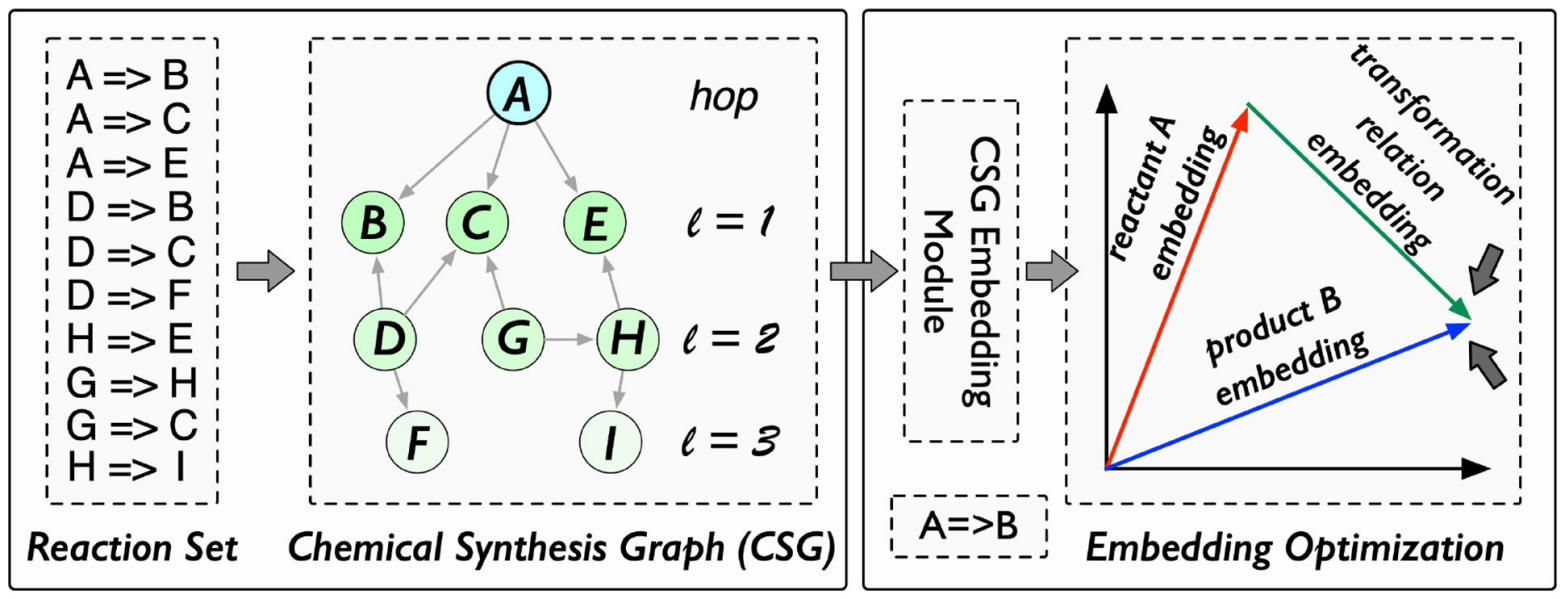
Left: an illustration of converting a chemical reaction set into a chemical synthesis graph (CSG) from *A*’s perspective. In the CSG, nodes and edges represent a reactant/product set and reaction transformation relations, respectively. Right: an description of embedding optimization in 2D space for chemical reaction *i*: $A\overset{i}{\to }B$.

Motivated by this gap, we introduce a chemical synthesis graph (CSG) for MRL. In CSG, nodes represent the molecule sets (i.e. the reactants/products on one side of a chemical reaction) and edges denote the reaction transformations that occur when reactants are converted into products (termed as the reaction transformations relation in this paper). Figure 1a shows an example of converting a reaction set into a CSG which can reveal molecule relationships and can be used for molecular modeling. For instance, node *A*’s first-order neighbors (*B*, *C*, and *E*) are products that can be synthesized from *A* via chemical reactions. Node *A*’s second-order neighbors (*D*, *G*, and *H*) indicate a property or structural similarity to *A*, evidenced by their shared reaction products. For example, both *A* and *G* can produce *C* through a specific reaction. Moreover, molecule *D* is likely more similar to *A* than either *G* or *H*, as it shares a larger number of reaction products with *A*. It can be observed that, compared to the RXGL method (Li *et al.* 2024), which constructs a reaction-aware graph using individual molecules, our strategy builds the CSG from the overall reactants/products. The CSG intuitively reflects the direct relationship between reactants and products in a chemical reaction, and it contains rich semantic information and structural relationships that aid in the MRL task.

**Figure 2.**
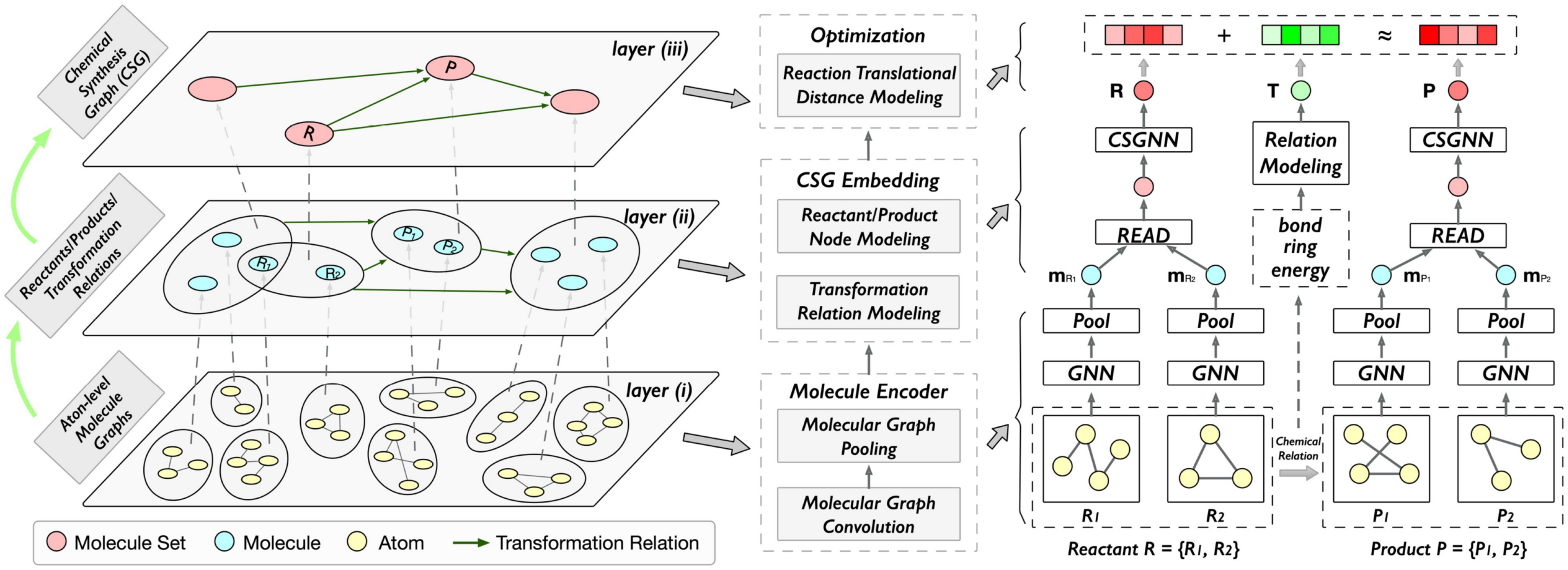
The framework of our model CSGL is structured in three layers. Left subgraph: Layer (i): Each node represents an atom, and the edges between atoms represent bonds. The larger circle indicates a molecule, which is a graph composed of atoms. We model the molecules at this layer using a molecule encoder (see Section 2.2.1). Layer (2): Each node represents a molecule, and the larger circle represents a collection of molecules corresponding to a reactant or product in a chemical reaction. The directed edges indicate the transformation from reactants to products. We model these reactants/products and their transformation relationships using the CSG embedding module (see Section 2.2.2). Layer (3): Each node represents a collection of molecules (reactant/product). This layer is used for optimizing the model parameters (see Section 2.2.3). Right subgraph: An example of a chemical reaction R→P, where $R=\{R_{1},R_{2}\}$ is the reactant and $P=\{P_{1},P_{2}\}$ is the product.

Based on the CSG, we propose a CSG learning (CSGL) framework, which has a hierarchical structure (see Fig. 2). In the lower level, we first use a molecule graph encoder to model the initial feature embeddings of molecules. Then, at the higher level, we design a chemical synthesis GNN (CSGNN) to refine the embeddings of molecule nodes (i.e. reactant/product set) by considering the connectivity in the CSG. To model the transformation relations (i.e. edges in the CSG), we consider three important features (bond changes, ring changes, and energy changes) in the reaction transformation process. Finally, inspired by RXGL (Li *et al.* 2024), CSGL introduces a reaction translational distance module to optimize node and edge embeddings. This process ensures that the sum of the reactant node embedding and reaction transformation relation embedding approximates the product node embedding for each link in the CSG, as shown in Fig. 1b. With this strategy, we not only model the changes occurring in reactions but also enhance the embeddings of molecules and the relations involved in reaction transformations.

Experimental results indicate that our CSGL can generate molecule embeddings that are effective for several downstream tasks (i.e. product prediction, chemical reaction classification, and molecular property prediction).

We summarize our contributions as follows:

- We propose a hierarchical framework CSGL, which enhances molecule representations by using the low-level molecular graph and high-level chemical reaction synthesis graph.
- We develop a CSGNN that leverages the idea of graph convolution to model the connectivity structure of the CSG, thereby refining molecular representations.
- We design a chemical reaction translational distance module that uses transformation relationships of chemical reactions to optimize embeddings of molecule node in the CSG.
- The effectiveness of our proposed model CSGL is validated by experiments across three downstream tasks.

## 2 Materials and methods

In this section, we first introduce the notations and formulate the problems. Then, we present our model CSGL and describe its training process. Finally, we analyze the model complexity.

### 2.1 Preliminaries

#### 2.1.1 Notations

Suppose we have a set of molecules M, where each molecule $m_{i}\in M$ corresponds to a graph structure $G_{m_{i}}=(V_{m_{i}},E_{m_{i}})$. Here, $V_{m_{i}}$ and $E_{m_{i}}$ denote the sets of nodes (atoms) and edges (bonds) for molecule $m_{i}$, respectively. In addition, each molecule also participates in chemical reactions. Given a set of reactions X, each reaction $x_{i}\in X$ establishes a transformation relation “$\overset{i}{\to }$” between a reactant set $R_{i}$ and a product set $P_{i}$, as $R_{i}\overset{i}{\to }P_{i}$, where $R_{i}$ and $P_{i}$, are defined as follows:


(1) $$R_{i}=\{m_{i,r_{1}},m_{i,r_{2}},\ldots ,m_{i,r_{\left|R_{i}\right|}}\}\subset M,$$

(2) $$P_{i}=\{m_{i,p_{1}},m_{i,p_{2}},\ldots ,m_{i,p_{\left|P_{i}\right|}}\}\subset M.$$

Based on chemical reaction set X, we construct a CSG $G_{s}=(V_{s},E_{s})$, where $V_{s}={\left\{R_{i}\right\}}_{i=1}^{\left|X\right|}\cup {\left\{P_{i}\right\}}_{i=1}^{\left|X\right|}$ and $E_{s}={\left\{\overset{i}{\to }\right\}}_{i=1}^{\left|X\right|}$ denote the sets of nodes (reactants/products) and edges (transformation relations), respectively.

#### 2.1.2 Problem formulation

For our method CSGL, the inputs include the graph structure of molecule $m_{i}$’s graph structure $G_{m_{i}}$, CSG $G_{s}$, and reaction set X, we aim to design a function, denoted as F, to learn the molecule representation, as follows: $m_{i}=F(m_{i}|G_{m_{i}},G_{s},X,\Theta )$, in which $m_{i}$ is $m_{i}$’s representation and Θ is the parameters of function F.

### 2.2 CSGL model

Figure 2 shows the framework of our method CSGL which has a hierarchical structure. Specifically, this structure consists of three key components arranged from bottom to top: layer (i) a molecule encoder that utilizes the molecular graph structures to learn embeddings of individual molecules; layer (ii) a CSG embedding module that utilizes chemical reaction information to model representations of nodes (reactants/products) and edges (reaction transformation relations) in the CSG; and layer (iii) a representation optimization module that leverages the CSG structure to optimize the embeddings of molecules and reaction transformation relations. In what follows, we provide a detailed introduction to these three modules.

#### 2.2.1 Molecule encoder

In this encoder, we first utilize GNNs (Kipf and Welling 2017, Li *et al.* 2022, 2025) to learn embeddings for atom nodes, and then apply the graph pooling operations on the molecular graph to generate molecule representations. Specifically, for molecule $m_{i}$ with its graph structure $G_{m_{i}}=(V_{m_{i}},E_{m_{i}})$, embedding $a_{j}^{k}$ of atom $a_{j}\in V_{m_{i}}$ in the *l*th GNN layer is modeled as follows:


(3) $$a_{j}^{l}=\text{AGG}(a_{k}^{l-1}|a_{k}\in N_{j}),$$

where $N_{j}$ represents the neighborhood of atom $a_{j}$ in graph $G_{m_{i}}$. AGG denotes the aggregation function used in GNNs. In this article, we implement function AGG using four GNNs [GCN (Kipf and Welling 2017), GAT (Veličković *et al.* 2018), SAGE (Hamilton *et al.* 2017), and TAG (Du *et al.* 2017)].

To represent initial feature $a_{j}^{0}$ of atom $a_{j}$, in this article, we utilize four atomic attributes: charge, element type, the number of connected hydrogen atoms, and whether the atom is in a ring. These attributes are each represented by a low-dimensional embedding (one-hot-encoding), and these feature embeddings are concatenated to form the initial atom feature. Note that we do not use explicit feature embeddings for bonds because the types of bonds can be inferred from the features of connected atoms (Wang *et al.* 2022a, Li *et al.* 2024).

After *L* layers of GNNs, we utilize a pooling function POOL to output the representation of molecule $m_{i}$, as follows:


(4) $$m_{i}=\text{POOL}(a_{j}^{L}|a_{j}\in V_{m_{i}}),$$

where $a_{j}^{L}$ is atom $a_{j}$’s representation after stacking *L* layers. The POOL function can be a simple permutation invariant function (e.g. summation, mean, and max) or a graph-level pooling strategy (Vinyals *et al.* 2015, Zhang *et al.* 2018).

Using a GNN on molecular graphs and pooling operations to obtain an overall molecular representation has been proven effective for molecular modeling (Wang *et al.* 2022a, Li *et al.* 2024). This is because molecules naturally exhibit a graph structure, as mentioned in Section 1. We will further analyze the impact of different graph convolution and pooling operations on performance in the experimental section.

#### 2.2.2 CSG embedding module

In the CSG, the nodes represent reactants/products and edges denote reaction transformations in chemical reactions. The CSG embedding module embeds these nodes and edges into a latent space, preparing for the subsequent representation optimization module.

1. Molecule node modeling

First, we introduce how to model the representations of nodes (reactant or product sets) in the CSG. Recalling that each reaction $x_{i}:R_{i}\overset{i}{\to }P_{i}$ involves a set of reactant molecules $R_{i}$ and product molecules $P_{i}$, we first use the molecular encoder in Section 2.2.1 to obtain representations for each molecule in $R_{i}$ and $P_{i}$, and then use a readout function READ and a CSGNN to derive representations for the $R_{i}$ and $P_{i}$ in graph $G_{s}$, as follows:


(5) $$R_{i}=\text{CSGNN}(\text{READ}(m_{j}|m_{j}\in R_{i}),G_{s}),$$

(6) $$P_{i}=\text{CSGNN}(\text{READ}(m_{k}|m_{k}\in P_{i}),G_{s}),$$

where $R_{i}$ and $P_{i}$ are the representations of reactant $R_{i}$ and product $P_{i}$, respectively. We define function READ using a summation operation (Wang *et al.* 2022a, Li *et al.* 2024):


(7) $$\text{READ}(m_{j}|m_{j}\in R_{i})=\sum_{m_{j}\in R_{i}}m_{j},$$

(8) $$\text{READ}(m_{k}|m_{k}\in P_{i})=\sum_{m_{k}\in P_{i}}m_{k}.$$

**Figure 3.**
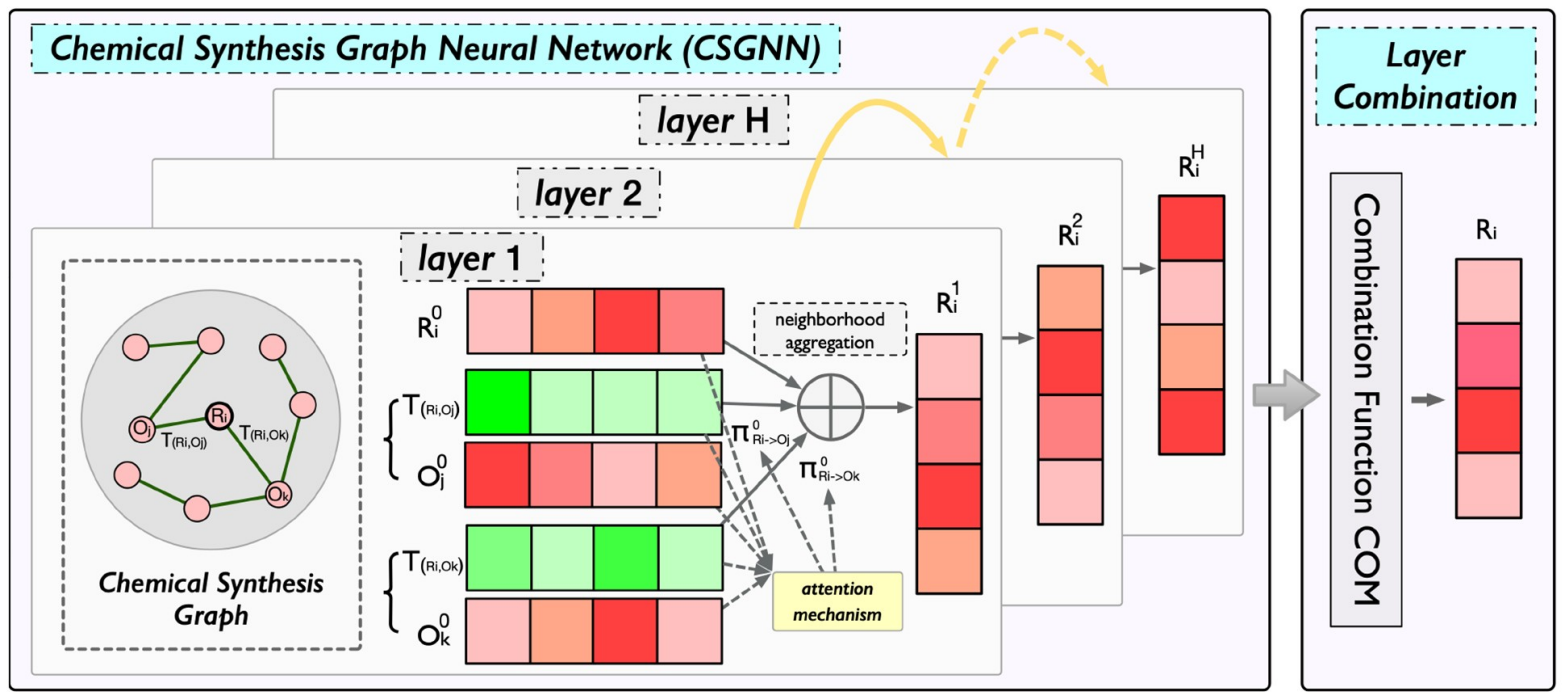
The illustration of chemical synthesis graph neural networks.

Then, we design a CSGNN to infuse connectivity information from the CSG into representations of reactant and product nodes, as shown in Fig. 3. CSGNN enhances the reactant and product representation modeling process through the neighborhood aggregation. Specifically, the *h*th aggregation is defined as:


(9) $$R_{i}^{h+1}=R_{i}^{h}+\sum_{O_{j}\in N_{R_{i}}}\pi_{R_{i}\to O_{j}}^{h+1}O_{j}^{h},$$

(10) $$P_{i}^{h+1}=P_{i}^{h}+\sum_{O_{k}\in N_{P_{i}}}\pi_{P_{i}\to O_{k}}^{h+1}O_{k}^{h},$$

where $N_{R_{i}}$ and $N_{P_{i}}$ are neighbor sets of node $R_{i}$ and $P_{i}$ in $G_{s}$; $R_{i}^{0}$, $P_{i}^{0}$, $O_{j}^{0}$, and $O_{k}^{0}$ are the 0th layer of the representations for nodes $R_{i}$, $P_{i}$, $O_{j}$, and $O_{k}$ in $G_{s}$, which are computed by using the READ function. The aggregation weight π between the central node and its neighbors is computed using an attention mechanism. For example, the computation of $\pi_{R_{i}\to O_{j}}^{h+1}$ is defined as follows:


(11) $$\pi_{R_{i}\to O_{j}}^{h+1}=\frac{\;\text{exp}(W_{a}^{h}(R_{i}^{h}⊙O_{j}^{h}⊙T_{\left(R_{i},O_{j}\right)}))}{\sum_{O_{j^{\prime }}\in N_{R_{i}}}\;\text{exp}\;(W_{a}^{h}(R_{i}^{h}⊙O_{j^{\prime }}^{h}⊙T_{\left(R_{i},O_{j^{\prime }}\right)}))},$$

where ⊙ is the element-wise product, $W_{a}$ is the weight matrix for the attention, and $T_{\left(R_{i},O_{j}\right)}$ is the reaction transformation relation between $R_{i}$ and $O_{j}$, calculated using Equation (16), which will be introduced later. After *H* times of aggregation, we combine embeddings obtained at each layer as reactant $R_{i}$’s representation $R_{i}$ and product $P_{i}$’s representation $P_{i}$, as:


(12) $$R_{i}=\frac{1}{H+1}\sum_{h\in [0,H]}R_{i}^{h},\;\;P_{i}=\frac{1}{H+1}\sum_{h\in [0,H]}P_{i}^{h}.$$

In this way, we incorporate CSG structural information to model the representations of reactant and product nodes.

2) Transformation relation modeling

Then, we explain how to model the representation of edges in the CSG. In real-world scenarios, reactions involve multiple transformations. For a reaction $x_{i}:R_{i}\overset{i}{\to }P_{i}$, we focus on modeling the edges (i.e. reaction transformation relation $\overset{i}{\to }$) in the CSG by considering three key features:

- Changes in the total number of bonds. The feature of this change is described by the difference in the total number of chemical bonds between reactants and products before and after the reaction, as follows:
  (13) $$T_{i,\text{bond}}=(\text{NB}(R_{i})-\text{NB}(P_{i}))\cdot e_{\text{bond}},$$
  where function NB is utilized to count the total number of chemical bonds in either the reactants (e.g. $R_{i}$) or products (e.g. $P_{i}$), and $e_{\text{bond}}$ serves as the feature embedding for the change in the total number of bonds.
- Changes in the total number of rings. The feature of this change is described by the difference in the total number of rings between reactants and products in the reaction, as:
  (14) $$T_{i,\text{ring}}=(\text{NR}(R_{i})-\text{NR}(P_{i}))\cdot e_{\text{ring}},$$
  where function NR is used to count the total ring number in reactants (e.g. $R_{i}$) and products (e.g. $P_{i}$), and $e_{\text{ring}}$ is the feature embedding for the change in the total ring number.
- Changes in energy of reactions (exothermic or endothermic). Chemical reactions can be categorized as either exothermic or endothermic. These energy changes can be modeled by analyzing the differences in the counts of various bond types, because the energy changes in reactions are primarily due to the breaking and forming of bonds (Sanderson 2012). In this work, we consider 15 common bond types, which are extracted utilizing RDKit (https://www.rdkit.org/), as detailed in the Supplementary Material, available as supplementary data at *Bioinformatics* online. In this way, we approximately describe the feature of energy changes in reactions by the differences in the counts of various chemical bonds between reactants and products in reactions, as follows:
  (15) $$T_{i,\text{energy}}\approx \sum_{b_{j}\in s(B)}({\;\text{NB}}_{b_{j}}(R_{i})-{\text{NB}}_{b_{j}}(P_{i}))\cdot e_{b_{j}},$$
  where s(B) is the set of bonds we consider in this work, function ${\text{NB}}_{b_{j}}$ is used to count the total number of bond $b_{j}$ in reactants (e.g. $R_{i}$) or products (e.g. $P_{i}$), and $e_{b_{j}}$ is the feature embedding for the change in the number of bond $b_{j}$.

After modeling the above three aspect features, we integrate them into the representation of the reaction transformation relation (i.e. $\overset{i}{\to }$ for $x_{i}:R_{i}\overset{i}{\to }P_{i}$), as follows:


(16) $$T_{i}=T_{i,\text{bond}}+T_{i,\text{ring}}+T_{i,\text{energy}}.$$

#### 2.2.3 Representation optimization module

**Figure 4.**
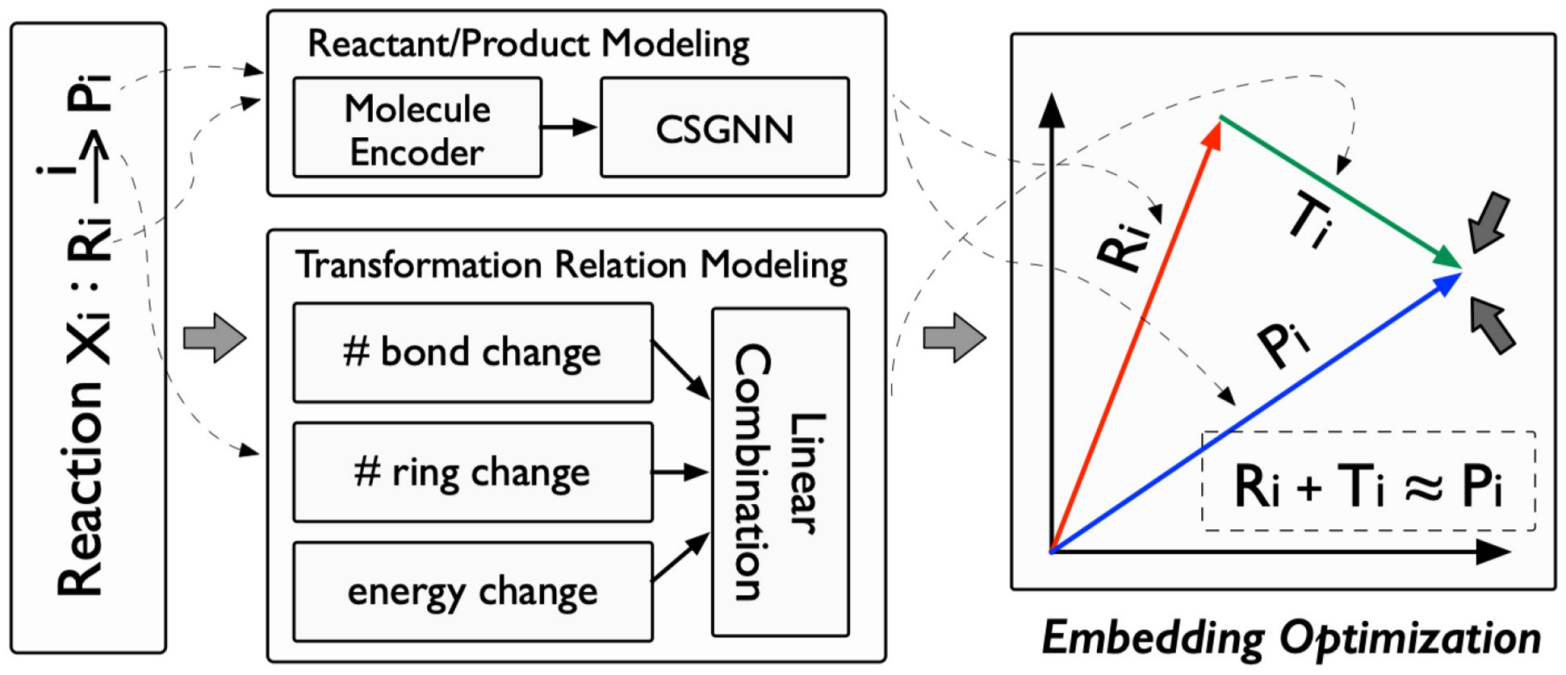
The optimization process of our proposed method.

As shown in Fig. 4, we use the idea (Li *et al.* 2024) of translational distance in reactions to optimize the embeddings of reactant and product nodes, and the chemical reaction transformation relations in the CSG. To be specific, for a given reaction $x_{i}:R_{i}\overset{i}{\to }P_{i}$, we model the change of the reaction as a translation from the reaction embedding to the product embedding; in other words, we impose that the product embedding approximates the sum of the reactant embedding and the transformation relation embedding, as follows:


(17) $$R_{i}+T_{i}\approx P_{i},$$

where $R_{i}$, $P_{i}$, $T_{i}$ are the embeddings of reactant $R_{i}$, product $P_{i}$, and relation $\overset{i}{\to }$ from the CSG embedding module.

To optimize the model, we employ the contrastive learning technique (Radford *et al.* 2021, Wang *et al.* 2022a). In a minibatch of reaction data $X_{B}$, we consider matched reactant–product pairs as positive pairs and minimize their embedding distances. Instead, unmatched pairs are identified as negative, and we maximize the embedding distances. We use the margin-based loss function (Bordes *et al.* 2013, Li *et al.* 2024):


(18) $$\begin{array}{c} L_{X}=\frac{1}{\left|X_{B}\right|}\sum_{x_{i}\in X_{B}}||R_{i}+T_{i}-P_{i}|{|}_{2}+\\ \;\;\;\;\;\frac{1}{\left|X_{B}{|}^{2}-|X_{B}\right|}\sum_{x_{i}\in X_{B}}\sum_{x_{j}\in X_{B}}\text{max}(\gamma -||R_{i}+T_{i}-P_{j}|{|}_{2},0), \end{array}$$

in which $x_{i}\neq x_{j}$ and γ>0 is a margin hyperparameter.

To train our methods, we define the final loss function as:


(19) $$L_{\mathrm{oss}}=L_{X}+\lambda ||\Theta |{|}_{F}^{2},$$

where $L_{X}$ is calculated from Equation (18), Θ is the model parameter, and λ controls the strength of L2-regularization.

## 3 Results

We first evaluate our CSGL performance on three downstream tasks. Then, we conduct ablation and hyperparameter analysis experiments to further analyze our method CSGL.

### 3.1 Product prediction

The product prediction task is widely used for analyzing molecular representations (Wang *et al.* 2022a, Li *et al.* 2024). This task involves predicting the products given the reactants.

**Dataset.** We use the USPTO-50K dataset (Dai *et al.* 2019, Li *et al.* 2024, Liu *et al.* 2024) for this task. This dataset contains 50 000 chemical reactions. Following Li *et al.* (2024), we randomly split the data into 80% for training, 10% for evaluation, and 10% for testing.

**Evaluation protocol.** Following Wang *et al.* (2022a) and Li *et al.* (2024), the product prediction task is considered as a ranking problem. First, we utilize $X_{\mathrm{test}}$ and $P_{\mathrm{test}}$ to denote the reaction set and their corresponding product set in the test set, respectively. Then, we leverage a matching function to calculate the match score between a reactant $R_{i}$ and each product $P_{j}\in P_{\mathrm{test}}$ in each chemical reaction $x\in X_{\mathrm{test}}$. Finally, we evaluate the effectiveness of models in the product prediction task by ranking of the score of correct product $P_{i}$. In this article, we employ *MRR* (mean reciprocal rank) and *Hit@K* (hit ratio at a cutoff value of *K*) as evaluation metrics.

**Baselines.** We compare CSGL with Mol2vec (Jaeger *et al.* 2018), MolBERT (Fabian *et al.* 2020), ChemBERTa-2 (Ahmad *et al.* 2022), MolR (Wang *et al.* 2022a), and RXGL (Li *et al.* 2024). For Mol2vec, MolBERT, and ChemBERTa-2, we first use them to generate reactant and product representations, and then apply the matching functions defined in Wang *et al.* (2022a) and Li *et al.* (2024) to define their matching function. For MolR and RXGL, we follow their article to define the matching function. For our CSGL, we define its matching function based on Equation (17), i.e. the smaller the distance between the sum of reactant embedding plus relation embedding and product embedding, the higher the matching score. For MolR, RXGL, and CSGL, four GNNs [i.e. GCN (Kipf and Welling 2017), GAT (Veličković *et al.* 2018), SAGE (Hamilton *et al.* 2017), TAG (Du *et al.* 2017)] are used as molecule graph encoders.

**Hyperparameter setting.** Our model CSGL is optimized by Adam optimizer (Kingma and Ba 2015). The learning rate is tuned in $\left\{10^{-5},10^{-4},10^{-3},10^{-2}\right\}$. We set the GNN layer number in the molecule encoder and CSGNN as 2 and 1. In addition, embedding size *d* and margin γ are selected from values of {32,64,128,256,512,1024} and {1,2,4,8,16}. Some designs and hyperparameters of our CSGL are analyzed in Section 3.4.

**Results.** From the results in Table 1, we find that our CSGL(TAG) generally achieves the best performance among different molecule graph encoders. This is likely due to TAG’s ability to capture multi-hop information through topology-aware aggregation, providing a broader contextual view than other architectures (GCN, GAT, and GraphSAGE), which mainly rely on first-order neighbors. In addition, we have two further observations. First, there exists a compatibility issue between certain molecule graph encoders and model architectures. For example, RXGL(SAGE) outperforms CSGL(SAGE), suggesting that SAGE is more compatible with RXGL’s design than with CSGL. Second, the choice of molecule graph encoder has a significant impact on overall performance. For instance, CSGL(GAT) underperforms compared to the MolR(TAG). This indicates that although CSGL is effective, its performance may be limited by the suboptimal representation quality of GAT for molecular graphs, showing the importance of selecting a suitable graph encoder.

**Table 1.** Results of product prediction on the USPTO-50K dataset.^a^

Methods	MRR	Hit@1	Hit@3	Hit@5
Mol2vec	0.835	0.801	0.858	0.874
MolBERT	0.913	0.874	0.942	0.961
ChemBERTa-2	0.833	0.776	0.867	0.901
MolR(GCN)	$0.958_{\left(0.003\right)}$	$0.944_{\left(0.006\right)}$	$0.966_{\left(0.003\right)}$	$0.971_{\left(0.005\right)}$
MolR(GAT)	$0.952_{\left(0.002\right)}$	$0.931_{\left(0.004\right)}$	$0.960_{\left(0.003\right)}$	$0.965_{\left(0.002\right)}$
MolR(SAGE)	$0.972_{\left(0.005\right)}$	$0.960_{\left(0.005\right)}$	$0.977_{\left(0.003\right)}$	$0.984_{\left(0.004\right)}$
MolR(TAG)	$0.974_{\left(0.010\right)}$	$0.965_{\left(0.009\right)}$	$0.981_{\left(0.007\right)}$	$0.989_{\left(0.005\right)}$
RXGL(GCN)	$0.965_{\left(0.007\right)}$	$0.954_{\left(0.002\right)}$	$0.972_{\left(0.003\right)}$	$0.977_{\left(0.004\right)}$
RXGL(GAT)	$0.967_{\left(0.009\right)}$	$0.958_{\left(0.011\right)}$	$0.973_{\left(0.006\right)}$	$0.978_{\left(0.007\right)}$
RXGL(SAGE)	$0.982_{\left(0.005\right)}$	$0.973_{\left(0.003\right)}$	$0.985_{\left(0.004\right)}$	$0.988_{\left(0.006\right)}$
RXGL(TAG)	$0.979_{\left(0.004\right)}$	$0.974_{\left(0.009\right)}$	$0.984_{\left(0.005\right)}$	$0.990_{\left(0.004\right)}$
CSGL(GCN)	$0.967_{\left(0.007\right)}$	$0.956_{\left(0.006\right)}$	$0.976_{\left(0.007\right)}$	$0.982_{\left(0.007\right)}$
CSGL(GAT)	$0.963_{\left(0.005\right)}$	$0.952_{\left(0.009\right)}$	$0.970_{\left(0.009\right)}$	$0.976_{\left(0.010\right)}$
CSGL(SAGE)	$0.979_{\left(0.004\right)}$	$0.973_{\left(0.006\right)}$	$0.984_{\left(0.006\right)}$	$0.987_{\left(0.004\right)}$
CSGL(TAG)	$0.984_{\left(0.006\right)}$	$0.979_{\left(0.007\right)}$	$0.989_{\left(0.005\right)}$	$0.989_{\left(0.005\right)}$

a The numbers in brackets are the standard deviations. Bold values denote the best values of all methods.

### 3.2 Reaction classification

In this section, we conduct experiments to analyze the effect of our methods on the chemical reaction classification task, which aims at predicting the category of a given reaction.

**Experiment setup.** In this task, we use two datasets: Schneider (Schneider *et al.* 2015, Li *et al.* 2024) and USPTO-MTL (Lu and Zhang 2022, Li *et al.* 2024). Specifically, the former contains 38 800 reactions with 46 classes and the latter dataset includes 143 535 reactions with 1000 classes. Following the setting in Li *et al.* (2024), each dataset is divided into 80% for training, 10% for evaluation, and 10% for testing. We compare our CSGL with MACCS (Cereto-Massagué *et al.* 2015), Mol2vec (Jaeger *et al.* 2018), MolBERT (Fabian *et al.* 2020), ChemBERTa-2 (Ahmad *et al.* 2022), MolR (Wang *et al.* 2022a), and RXGL (Li *et al.* 2024). Due to the differences in the CSG structure between the pre-training phase and reaction classification task, for our CSGL, we train a variant without the CSGNN on the USPTO-50K dataset. We then use the pre-trained model to obtain reactant and product embeddings for each reaction in the reaction classification task. The reaction feature is created by concatenating reactant and product embeddings. Finally, we use an MLP as the prediction decoder. We use accuracy, precision, and recall as metrics.

**Results.**  Table 2 shows the results. Some observations are similar to those in the product prediction experiments and are omitted here for brevity. Overall, our CSGL outperforms baselines in various GNN backbones. For instance, CSGL(TAG) improves over the strongest baselines w.r.t. accuracy by 2.43% and 4.58% on the Schneider and USPTO-MTL datasets. These findings indicate that the molecule embeddings learned by our CSGL can be transferred to the reaction classification task.

**Table 2.** Reaction classification results.^a^

Methods	Schneider			USPTO-MTL		
	Accuracy	Precision	Recall	Accuracy	Precision	Recall
MACCS	$0.906_{\left(0.010\right)}$	$0.912_{\left(0.012\right)}$	$0.908_{\left(0.015\right)}$	$0.848_{\left(0.012\right)}$	$0.880_{\left(0.009\right)}$	$0.803_{\left(0.013\right)}$
Mol2vec	$0.856_{\left(0.012\right)}$	$0.864_{\left(0.013\right)}$	$0.850_{\left(0.009\right)}$	$0.751_{\left(0.016\right)}$	$0.728_{\left(0.017\right)}$	$0.629_{\left(0.014\right)}$
MolBERT	$0.849_{\left(0.014\right)}$	$0.852_{\left(0.020\right)}$	$0.847_{\left(0.012\right)}$	$0.738_{\left(0.009\right)}$	$0.692_{\left(0.007\right)}$	$0.583_{\left(0.008\right)}$
ChemBERTa-2	$0.876_{\left(0.008\right)}$	$0.878_{\left(0.011\right)}$	$0.875_{\left(0.010\right)}$	$0.885_{\left(0.006\right)}$	$0.880_{\left(0.008\right)}$	$0.857_{\left(0.008\right)}$
MolR(GCN)	$0.879_{\left(0.013\right)}$	$0.885_{\left(0.010\right)}$	$0.882_{\left(0.013\right)}$	$0.853_{\left(0.020\right)}$	$0.873_{\left(0.018\right)}$	$0.813_{\left(0.015\right)}$
MolR(GAT)	$0.870_{\left(0.024\right)}$	$0.877_{\left(0.026\right)}$	$0.873_{\left(0.021\right)}$	$0.862_{\left(0.017\right)}$	$0.886_{\left(0.015\right)}$	$0.834_{\left(0.014\right)}$
MolR(SAGE)	$0.882_{\left(0.030\right)}$	$0.891_{\left(0.027\right)}$	$0.881_{\left(0.027\right)}$	$0.874_{\left(0.023\right)}$	$0.897_{\left(0.023\right)}$	$0.839_{\left(0.026\right)}$
MolR(TAG)	$0.891_{\left(0.025\right)}$	$0.898_{\left(0.030\right)}$	$0.895_{\left(0.026\right)}$	$0.888_{\left(0.024\right)}$	$0.900_{\left(0.023\right)}$	$0.852_{\left(0.024\right)}$
RXGL(GCN)	$0.887_{\left(0.016\right)}$	$0.893_{\left(0.017\right)}$	$0.889_{\left(0.017\right)}$	$0.875_{\left(0.018\right)}$	$0.873_{\left(0.017\right)}$	$0.849_{\left(0.015\right)}$
RXGL(GAT)	$0.872_{\left(0.026\right)}$	$0.879_{\left(0.022\right)}$	$0.874_{\left(0.020\right)}$	$0.873_{\left(0.019\right)}$	$0.870_{\left(0.018\right)}$	$0.836_{\left(0.016\right)}$
RXGL(SAGE)	$0.907_{\left(0.029\right)}$	$0.897_{\left(0.026\right)}$	$0.906_{\left(0.025\right)}$	$0.889_{\left(0.014\right)}$	$0.896_{\left(0.015\right)}$	$0.858_{\left(0.018\right)}$
RXGL(TAG)	$0.899_{\left(0.033\right)}$	$0.909_{\left(0.029\right)}$	$0.901_{\left(0.031\right)}$	$0.895_{\left(0.019\right)}$	$0.892_{\left(0.016\right)}$	$0.867_{\left(0.018\right)}$
CSGL(GCN)	$0.909_{\left(0.015\right)}$	$0.911_{\left(0.015\right)}$	$0.910_{\left(0.020\right)}$	$0.911_{\left(0.022\right)}$	$0.912_{\left(0.025\right)}$	$0.894_{\left(0.023\right)}$
CSGL(GAT)	$0.876_{\left(0.025\right)}$	$0.881_{\left(0.024\right)}$	$0.878_{\left(0.022\right)}$	$0.895_{\left(0.025\right)}$	$0.895_{\left(0.026\right)}$	$0.862_{\left(0.020\right)}$
CSGL(SAGE)	$0.902_{\left(0.013\right)}$	$0.905_{\left(0.016\right)}$	$0.903_{\left(0.019\right)}$	$0.924_{\left(0.019\right)}$	$0.930_{\left(0.020\right)}$	$0.898_{\left(0.020\right)}$
CSGL(TAG)	$0.929_{\left(0.022\right)}$	$0.931_{\left(0.028\right)}$	$0.930_{\left(0.027\right)}$	$0.936_{\left(0.021\right)}$	$0.939_{\left(0.025\right)}$	$0.918_{\left(0.021\right)}$

a The numbers in brackets are the standard deviations. Bold values are the best values of all methods.

### 3.3 Molecular property prediction

The goal of this task is to predict molecular property labels, which is a popular evaluation method for assessing molecule representations (Wang *et al.* 2022a,c, Li *et al.* 2024).

**Datasets.** We use five datasets (Wu *et al.* 2018): BBBP, BACE, Tox21, ClinTox, HIV. They are widely utilized in the molecular property prediction task (Wang *et al.* 2022a,c).

**Baselines.** We use four group baselines: (i) SMILES-based: ChemBERTa (Chithrananda *et al.* 2020) and MolBERT (Fabian *et al.* 2020); (ii) Fingerprint-based: Mol2vec (Jaeger *et al.* 2018), ECFP4 (Rogers and Hahn 2010), GraphConv (Duvenaud *et al.* 2015), Weave (Kearnes *et al.* 2016), D-MPNN (Yang *et al.* 2019), CDDD (Winter *et al.* 2019); (iii) GNN-based: GraphCL (You *et al.* 2020), GraphLoG (Xu *et al.* 2021); and (iv) Reaction-enhanced: MolR (Wang *et al.* 2022a), ReaKE (Wang *et al.* 2022c), RXGL (Li *et al.* 2024).

**Experiment setting.** Following Wang *et al.* (2022a) and Li *et al.* (2024), each dataset is split into training, validation, and test sets in an 8:1:1 ratio. For CSGL, since the property prediction task does not involve the CSG structure, we use the same method in the reaction classification task to obtain molecule embeddings. These embeddings are then input into a logistic regression for prediction. AUC is used as the metric.

**Results.**  Table 3 shows the results. The baseline results are taken from Wang *et al.* (2022a,c) and Li *et al.* (2024). We find that our CSGL performs the best on four out of five datasets, indicating that learned molecular embeddings are beneficial for molecule-related tasks. We further add molecular embedding analysis in the Supplementary Material, available as supplementary data at *Bioinformatics* online.

**Table 3.** Results of molecular property prediction on five datasets.^a^

Method		BBBP	BACE	Tox21	ClinTox	HIV
SMILES-based methods	ChemBERTa^★^	0.643	–	0.728	0.733	0.622
	MolBERT^★^	$0.762_{\left(0.000\right)}$	$0.866_{\left(0.000\right)}$	–	–	$0.783_{\left(0.000\right)}$
Fingerprint-based methods	Mol2vec^★^	$0.872_{\left(0.021\right)}$	$0.862_{\left(0.027\right)}$	$0.803_{\left(0.041\right)}$	$0.841_{\left(0.062\right)}$	$0.769_{\left(0.021\right)}$
	ECFP4^★^	0.729	0.867	0.822	0.799	0.792
	GraphConv^★^	0.690	0.783	0.829	0.807	0.763
	Weave^★^	0.671	0.806	0.820	0.832	0.703
	D-MPNN^★^	0.708	–	0.688	0.906	0.752
	CDDD^★^	$0.761_{\left(0.000\right)}$	$0.833_{\left(0.000\right)}$	–	–	$0.753_{\left(0.00\right)}$
GNN-based methods	GraphCL^★^	$0.695_{\left(0.005\right)}$	$0.782_{\left(0.012\right)}$	$0.754_{\left(0.009\right)}$	$0.701_{\left(0.019\right)}$	$0.776_{\left(0.009\right)}$
	GraphLoG^★^	$0.725_{\left(0.008\right)}$	$0.835_{\left(0.012\right)}$	$0.757_{\left(0.005\right)}$	$0.767_{\left(0.033\right)}$	$0.778_{\left(0.008\right)}$
Reaction-enhanced methods	MolR^★^	$0.890_{\left(0.032\right)}$	$0.882_{\left(0.019\right)}$	$0.818_{\left(0.023\right)}$	$0.916_{\left(0.039\right)}$	$0.802_{\left(0.024\right)}$
	ReaKE^♠^	–	0.898	0.824	0.874	0.817
	RXGL^♣^	$0.901_{\left(0.024\right)}$	$0.906_{\left(0.017\right)}$	$0.852_{\left(0.026\right)}$	$0.921_{\left(0.030\right)}$	–
Our method	CSGL	$0.908_{\left(0.019\right)}$	$0.892_{\left(0.024\right)}$	$0.864_{\left(0.027\right)}$	$0.924_{\left(0.028\right)}$	$0.822_{\left(0.023\right)}$

a The numbers in brackets are the standard deviations. The results of symbols ★, ♠, and ♣ are taken from MolR (Wang *et al.* 2022a), ReaKE (Wang *et al.* 2022c), and RXGL (Li *et al.* 2024), respectively. Bold values denote the best values of all methods.

### 3.4 Detailed model analysis

In this subsection, we conduct the model analysis of our CSGL.

#### 3.4.1 Pooling operation sensitivity

We take our CSGL(GCN) as an example to study the impact of graph pooling operations [Equation (4)] on model performance. Specifically, we consider six pooling models: (i) SumPooling, which is what we use in our methods; (ii) AvgPooling, which uses average pooling on atoms in the molecular graph; (iii) MaxPooling, which uses max pooling on atoms in the molecular graph; (iv) Attention, which uses an MLP-based attention mechanism to weight the summation of atom embeddings as the molecule embedding; (v) SortPooling (Zhang *et al.* 2018), which first sorts atom embeddings and then calculates the molecule embedding based on sorting; (vi) Set2set (Vinyals *et al.* 2015), which first calculates a query vector and then uses this vector to aggregate atom embeddings as the molecule embedding. The results in Table 4 shows that SumPooling outperforms other poolings, which suggests that this operation is a simple and effective way to model molecular graphs.

**Table 4.** POOL function analysis on the USPTO-50K dataset.^a^

Methods	MRR	Hit@1	Hit@3	Hit@5
SumPooling	$0.967_{\left(0.007\right)}$	$0.956_{\left(0.006\right)}$	$0.976_{\left(0.007\right)}$	$0.982_{\left(0.007\right)}$
AvgPooling	$0.906_{\left(0.005\right)}$	$0.880_{\left(0.006\right)}$	$0.921_{\left(0.005\right)}$	$0.935_{\left(0.004\right)}$
MaxPooling	$0.895_{\left(0.005\right)}$	$0.871_{\left(0.007\right)}$	$0.909_{\left(0.005\right)}$	$0.924_{\left(0.006\right)}$
Attention	$0.918_{\left(0.007\right)}$	$0.898_{\left(0.007\right)}$	$0.932_{\left(0.006\right)}$	$0.941_{\left(0.006\right)}$
Sortpooling	$0.770_{\left(0.010\right)}$	$0.705_{\left(0.011\right)}$	$0.813_{\left(0.008\right)}$	$0.849_{\left(0.008\right)}$
Set2Set	$0.889_{\left(0.011\right)}$	$0.860_{\left(0.009\right)}$	$0.907_{\left(0.007\right)}$	$0.919_{\left(0.007\right)}$

a Bold values denote the best values of all methods.

#### 3.4.2 Effect of chemical synthesis graph neural network

**Figure 5.**
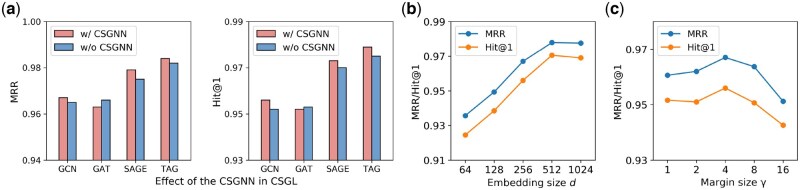
Effect of the CSGNN and hyperparameter study on the USPTO-50K dataset.

In Section 2.2.2, we design a CSGNN to model molecule representations in the synthesis graph. This subsection conducts the ablation study to analyze CSGNN’s effectiveness. Figure 5a compares the performance on the product prediction with and without the CSGNN (i.e. w/and w/o CSGNN). We find that in the product prediction task, CSGNN generally improves performance. However, in some cases, the improvement is not significant. One possible reason is that the dataset is sparse, which limits the connectivity of the graph structure. In future work, we plan to validate the effectiveness of this design on other datasets.

#### 3.4.3 Hyperparameter study

We study two hyperparameters: embedding size *d* and margin γ. The results of their sensitivity are shown in Fig. 5b and c. We find: (i) As *d* increases, performance initially improves but then starts to decline, likely due to overfitting. (ii) Margin size γ affects model training. Too large or too small γ results in unsatisfactory performance. We recommend setting γ to 4.

## 4 Conclusion and future work

In this article, we propose a CSGL framework to enhance MRL by incorporating chemical reactions as side information. Specifically, CSGL constructs a CSG where nodes represent sets of molecules either as reactants or products and edges represent transformation relations in reactions. Then, CSGL framework introduces a CSG embedding module to learn the embeddings of the reactant and product nodes, and their transformation relations in the CSG. In this module, we design a CSGNN to refine node representations in the CSG by considering their neighborhood. Finally, an optimization module is developed to optimize these embeddings by considering their reaction transformation distance. Extensive experimental results demonstrate that our proposed CSGL achieves strong performance across various downstream tasks, including product prediction, reaction classification, and molecular property prediction.

We plan to improve our model from (i) Molecular modeling: with our molecular graph encoder, our model can theoretically generate embeddings for any compound. In future work, we will design advanced data augmentation methods to enhance the encoder, improving representations for compounds with novel structures or lacking explicit reaction records. (ii) Relation modeling: we currently use bond-type count variations to capture energy changes in reactions, which help model transformation relations. Moving forward, we will incorporate additional reaction-related auxiliary information to better represent energy changes in chemical processes.

## Supplementary Material

btaf355_Supplementary_Data

## Data Availability

No new data were generated or analyzed in support of this research.

## References

[btaf355-B1] Ahmad W , Simon E, Chithrananda S et al ChemBERTa-2: towards chemical foundation models. arXiv, arXiv:2209.01712, 2022, preprint: not peer reviewed.

[btaf355-B2] Bordes A , Usunier N, Garcia-Duran A et al Translating embeddings for modeling multi-relational data. In: *Conference on Neural Information Processing Systems, USA* 26. USA: Curran Associates, 2013, 2787–95.

[btaf355-B3] Cereto-Massagué A , Ojeda MJ, Valls C et al Molecular fingerprint similarity search in virtual screening. Methods 2015;71:58–63. 25132639 10.1016/j.ymeth.2014.08.005

[btaf355-B4] Chithrananda S , Grand G, Ramsundar B. ChemBERTa: large-scale self-supervised pretraining for molecular property prediction. arXiv, arXiv:2010.09885, 2020, preprint: not peer reviewed.

[btaf355-B5] Dai H , Li C, Coley C et al Retrosynthesis prediction with conditional graph logic network. In: *Conference on Neural Information Processing Systems, Canada*. Vol. 32. USA: Curran Associates, 2019, 8870–80.

[btaf355-B6] Devlin J , Chang M-W, Lee K et al BERT: pre-training of deep bidirectional transformers for language understanding. arXiv, arXiv:1810.04805, 2018, preprint: not peer reviewed.

[btaf355-B7] Ding Z , Zhang T, Li Y et al RingFormer: a ring-enhanced graph transformer for organic solar cell property prediction. In: Association for the Advancement of Artificial Intelligence, Vol. 39. USA: AAAI Press, 2025, 155–63.

[btaf355-B8] Du J , Zhang S, Wu G et al Topology adaptive graph convolutional networks. arXiv, arXiv:1710.10370, 2017, preprint: not peer reviewed.

[btaf355-B9] Duvenaud D , Maclaurin D, Aguilera-Iparraguirre J et al Convolutional networks on graphs for learning molecular fingerprints. In: Conference on Neural Information Processing Systems, Canada. USA: Curran Associates, 2015, 2224–32.

[btaf355-B10] Fabian B , Edlich T, Gaspar H et al Molecular representation learning with language models and domain-relevant auxiliary tasks. arXiv, arXiv:2011.13230, 2020, preprint: not peer reviewed.

[btaf355-B11] Guo Z , Guo K, Nan B et al Graph-based molecular representation learning. arXiv, arXiv:2207.04869, 2022, preprint: not peer reviewed.

[btaf355-B12] Hamilton WL , Ying R, Leskovec J. Inductive representation learning on large graphs. In: Conference on Neural Information Processing Systems, USA. USA: Curran Associates. 2017, 1025–35.

[btaf355-B13] Jaeger S , Fulle S, Turk S. Mol2vec: unsupervised machine learning approach with chemical intuition. J Chem Inf Model 2018;58:27–35. 29268609 10.1021/acs.jcim.7b00616

[btaf355-B14] Kearnes S , McCloskey K, Berndl M et al Molecular graph convolutions: moving beyond fingerprints. J Comput Aided Mol Des 2016;30:595–608. 27558503 10.1007/s10822-016-9938-8 PMC5028207

[btaf355-B15] Kingma DP , Ba J. Adam: a method for stochastic optimization. In: International Conference on Learning Representations, USA. USA: Curran Associates, 2015, 1.

[btaf355-B16] Kipf TN , Welling M. Semi-supervised classification with graph convolutional networks. In: International Conference on Learning Representations, France. USA: Curran Associates, 2017, 1.

[btaf355-B17] Li A , Yang B, Huo H et al Hypercomplex graph collaborative filtering. In: The Web Conference, France. USA: ACM, 2022, 1914–22.

[btaf355-B18] Li A , Casiraghi E, Rousu J. Chemical reaction enhanced graph learning for molecule representation. Bioinformatics 2024;40:btae558. 39271156 10.1093/bioinformatics/btae558 PMC11639130

[btaf355-B19] Li A , Yang B, Huo H et al Self-supervised dual graph learning for recommendation. Knowl-Based Syst 2025;310:112967.

[btaf355-B20] Liu J , Yan C, Yu Y et al MARS: a motif-based autoregressive model for retrosynthesis prediction. Bioinformatics 2024;40:1. 10.1093/bioinformatics/btae115 PMC10948277 38426338

[btaf355-B21] Lu J , Zhang Y. Unified model for multitask reaction predictions with explanation. J Chem Inf Model 2022;62:1376–87. 35266390 10.1021/acs.jcim.1c01467 PMC8960360

[btaf355-B22] Mahmood O , Mansimov E, Bonneau R et al Masked graph modeling for molecule generation. Nat Commun 2021;12:3156. 34039973 10.1038/s41467-021-23415-2 PMC8155025

[btaf355-B23] Nguyen T , Torres-Flores T, Hwang C et al GLaD: synergizing molecular graphs and language descriptors for enhanced power conversion efficiency prediction in organic photovoltaic devices. In: The Conference on Information and Knowledge Management, USA. USA: ACM, 2024, 4777–85.

[btaf355-B24] Radford A , Kim JW, Hallacy C et al Learning transferable visual models from natural language supervision. In: *International Conference on Machine Learning, Virtual Event, PMLR.* USA: Curran Associates, 2021, 8748–63.

[btaf355-B25] Rogers D , Hahn M. Extended-connectivity fingerprints. J Chem Inf Model 2010;50:742–54. 20426451 10.1021/ci100050t

[btaf355-B26] Sanderson R. Chemical Bonds and Bonds Energy, Vol. 21. USA: Elsevier, 2012.

[btaf355-B27] Schneider N , Lowe DM, Sayle RA et al Development of a novel fingerprint for chemical reactions and its application to large-scale reaction classification and similarity. J Chem Inf Model 2015;55:39–53. 25541888 10.1021/ci5006614

[btaf355-B28] Segler MH , Preuss M, Waller MP. Planning chemical syntheses with deep neural networks and symbolic AI. Nature 2018;555:604–10. 29595767 10.1038/nature25978

[btaf355-B29] Su B , Du D, Yang Z et al A molecular multimodal foundation model associating molecule graphs with natural language. arXiv, arXiv:2209.05481, 2022, preprint: not peer reviewed.

[btaf355-B30] Vaswani A , Shazeer N, Parmar N et al Attention is all you need. In: Conference on Neural Information Processing Systems, USA. USA: Curran Associates, 2017, 5998–6008.

[btaf355-B31] Veličković P , Cucurull G, Casanova A et al Graph attention networks. In: International Conference on Learning Representations, Canada. USA: Curran Associates, 2018, 1.

[btaf355-B32] Vinyals O , Bengio S, Kudlur M. Order matters: sequence to sequence for sets. arXiv, arXiv:1511.06391, 2015, preprint: not peer reviewed.

[btaf355-B33] Wang H , Li W, Jin X et al Chemical-reaction-aware molecule representation learning. In: International Conference on Learning Representations, Virtual Event. USA: Curran Associates, 2022a, 1.

[btaf355-B34] Wang Y , Wang J, Cao Z et al Molecular contrastive learning of representations via graph neural networks. Nat Mach Intell 2022b;4:279–87.

[btaf355-B35] Wang Y , Zheng S, Rao J et al ReaKE: contrastive molecular representation learning with chemical synthetic knowledge graph. arXiv, 2022c, preprint: not peer reviewed. 10.1021/acs.jcim.4c00157 38484468

[btaf355-B36] Winter R , Montanari F, Noé F et al Learning continuous and data-driven molecular descriptors by translating equivalent chemical representations. Chem Sci 2019;10:1692–701. 30842833 10.1039/c8sc04175j PMC6368215

[btaf355-B37] Wu Z , Ramsundar B, Feinberg EN et al MoleculeNet: a benchmark for molecular machine learning. Chem Sci 2018;9:513–30. 29629118 10.1039/c7sc02664a PMC5868307

[btaf355-B38] Xu M , Wang H, Ni B et al Self-supervised graph-level representation learning with local and global structure. In: *International Conference on Machine Learning, Virtual Event, PMLR*. 2021, 11548–58. Curran Associates, USA.

[btaf355-B39] Yang K , Swanson K, Jin W et al Are learned molecular representations ready for prime time? arXiv, arXiv:1904.01561, 2019, preprint: not peer reviewed.

[btaf355-B40] You Y , Chen T, Sui Y et al Graph contrastive learning with augmentations. In: *Conference on Neural Information Processing Systems, Virtual Event*, Vol. 33. USA: Curran Associates, 2020, 5812–23.

[btaf355-B41] Zhang M , Cui Z, Neumann M et al An end-to-end deep learning architecture for graph classification. In: Association for the Advancement of Artificial Intelligence, USA, Vol. 32. AAAI Press, 2018, 4438–45.

