# Supplementary material for: CSGL: chemical synthesis graph learning for molecule representation
Source: Bioinformatics. 2025 Jun 19;41(7):btaf355. doi: 10.1093/bioinformatics/btaf355 (PMC12234999; doi:10.1093/bioinformatics/btaf355)
Supplement: btaf355_Supplementary_Data [file btaf355_supplementary_data.pdf]

Supplementary Material: CSGL: Chemical Synthesis Graph Learning for Molecule Representation

Anchen Li[1], Elena Casiraghi[1,2,3,4], and Juho Rousu[1]
1.Department of Computer Science, Aalto University, Finland; 2.AnacletoLab, Dipartimento di Informatica, University of Milan, Italy; 3.Environmental Genomics and Systems Biology Division, Lawrence Berkeley National Laboratory, Berkeley, CA, USA; 4.ELLIS, European Laboratory for Learning and Intelligent Systems, Milan Unit, Italy
anchen.li@aalto.fi

In the supplementary data, we introduce some details which have been omitted in the paper.

A. Chemical Bond Types

In this work, we consider fifteen common bond types, which are extracted utilizing RDKit (https://www.rdkit.org/), as detailed in the Table 1.

B. Molecule Embedding Analysis

We select BBBP dataset for our experiments, and CSGL(GCN) outputs the molecule embeddings. To visualize embeddings, we use t-SNE. Specifically, we consider eight molecular properties, including permeability, size (i.e., the number of non-hydrogen atoms), smallest rings (i.e., the number of rings), and five functional groups (i.e., carboxyl, tert-butyl, hydroxy, carbonyl, and amide groups). These five functional groups are chosen because of their high frequency in the dataset. From the results in Figure 1, we have the following findings: (1) Figure 1(a) shows molecules colored by their permeability property and we observe that two to three clusters represent molecules without the permeable property. (2) In Figures 1(b) $\sim$ 1(d) and 1(i) $\sim$ 1(l), molecules are colored based on their size, ring number, and functional group properties, respectively. We find that the embedding space can distinguish molecules based on these properties. We further conduct clustering analysis by using K-means method to cluster

Table 1: Illustration of fifteen bond types.

Symbol	Description
C-C	Carbon-Carbon Single Bond
C=C	Carbon-Carbon Double Bond
C#C	Carbon-Carbon Triple Bond
C-O	Carbon-Oxygen Single Bond
C=O	Carbon-Oxygen Double Bond
C-N	Carbon-Nitrogen Single Bond
C=N	Carbon-Nitrogen Double Bond
C#N	Carbon-Nitrogen Triple Bond
C-S	Carbon-Sulfur Single Bond
C-P	Carbon-Phosphorus Single Bond
C-Si	Carbon-Silicon Single Bond
C-F	Carbon-Fluorine Single Bond
C-Cl	Carbon-Chlorine Single Bond
C-Br	Carbon-Bromine Single Bond
C-I	Carbon-Iodine Single Bond

the molecule embeddings into eight clusters. The results are shown in Figures 1(e) $\sim$ 1(h) and 1(m) $\sim$ 1(p). Then, the percentage of molecules with different labels, relative to the total number of molecules in each cluster, is calculated. For instance, in cluster 7 of Figure 1(e), 83.7% of the molecules are labeled with *permeability*. Generally, a dominant label (i.e., a label with a high proportion) can be observed in each cluster. This indicates that the learned molecule embeddings successfully cluster molecules with similar property labels.

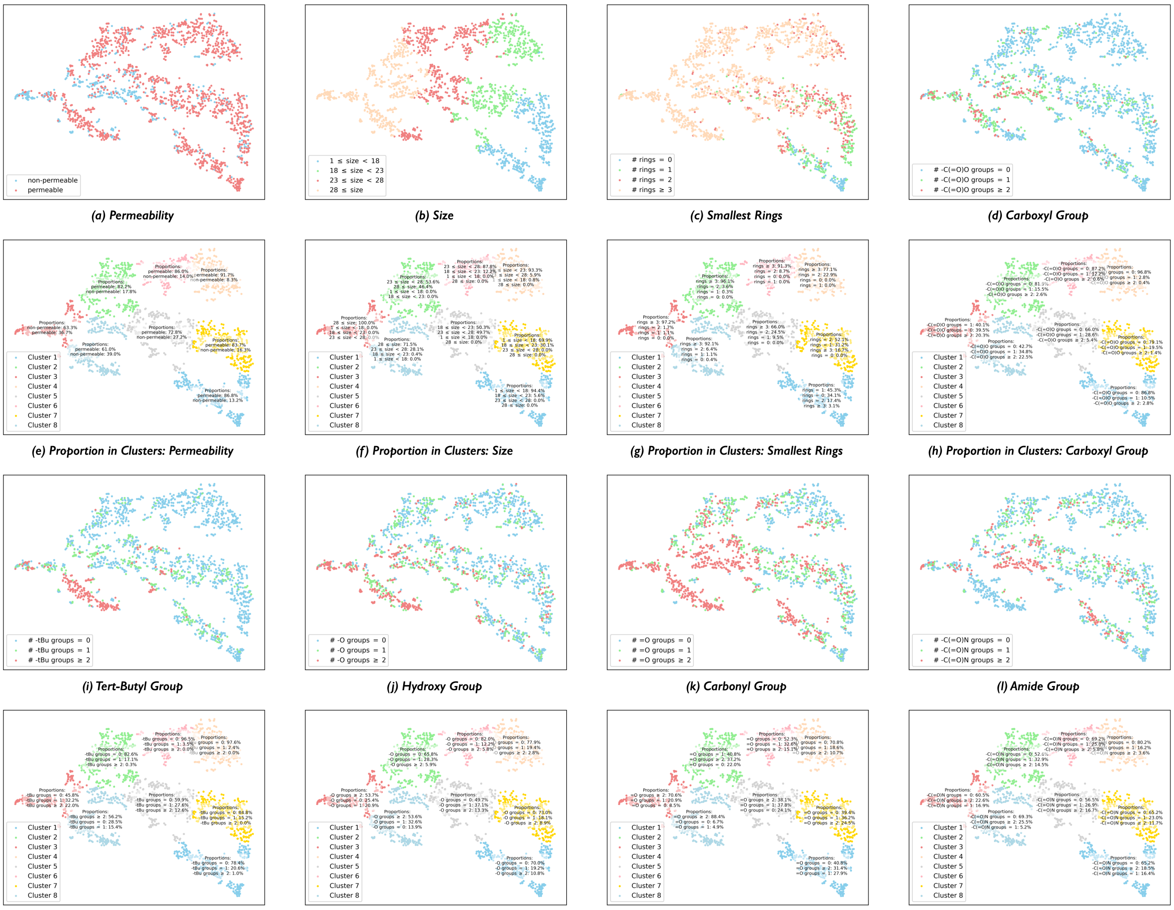

(a) Permeability (b) Size (c) Smallest Rings (d) Carboxyl Group

(e) Proportion in Clusters: Permeability (f) Proportion in Clusters: Size (g) Proportion in Clusters: Smallest Rings (h) Proportion in Clusters: Carboxyl Group

(i) Tert-Butyl Group (j) Hydroxy Group (k) Carbonyl Group (l) Amide Group

(m) Proportion in Clusters: Tert-Butyl Group (n) Proportion in Clusters: Hydroxy Group (o) Proportion in Clusters: Carbonyl Group (p) Proportion in Clusters: Amide Group

Figure 1: Visualized and cluster analysis for embeddings of molecules on the BBBP dataset w.r.t. molecular properties.
